# J-domain proteins: From molecular mechanisms to diseases

**DOI:** 10.1016/j.cstres.2023.12.002

**Published:** 2023-12-23

**Authors:** Jaroslaw Marszalek, Paolo De Los Rios, Douglas Cyr, Matthias P. Mayer, Vasista Adupa, Claes Andréasson, Gregory L. Blatch, Janice E.A. Braun, Jeffrey L. Brodsky, Bernd Bukau, J. Paul Chapple, Charlotte Conz, Sébastien Dementin, Pierre Genevaux, Olivier Genest, Pierre Goloubinoff, Jason Gestwicki, Colin M. Hammond, Justin K. Hines, Koji Ishikawa, Lukasz A. Joachimiak, Janine Kirstein, Krzysztof Liberek, Dejana Mokranjac, Nadinath Nillegoda, Carlos H.I. Ramos, Mathieu Rebeaud, David Ron, Sabine Rospert, Chandan Sahi, Reut Shalgi, Bartlomiej Tomiczek, Ryo Ushioda, Elizaveta Ustyantseva, Yihong Ye, Maciej Zylicz, Harm H. Kampinga

**Affiliations:** 1 Intercollegiate Faculty of Biotechnology, University of Gdansk and Medical University of Gdansk, Abrahama 58, Gdansk 80-307, Poland; 2 Institute of Physics, School of Basic Sciences, École Polytechnique Fédérale de Lausanne – EPFL, Lausanne CH 1015, Switzerland; 3 Institute of Bioengineering, School of Life Sciences, École Polytechnique Fédérale de Lausanne – EPFL, Lausanne CH 1015, Switzerland; 4 Department of Cell Biology and Physiology, School of Medicine, University of North Carolina at Chapel Hill, Chapel Hill, NC 27599, USA; 5 Center for Molecular Biology of Heidelberg University (ZMBH), Heidelberg 69120, Germany; 6 Zernike Institute for Advanced Materials, University of Groningen, Groningen, The Netherlands; 7 Department of Molecular Biosciences, The Wenner-Gren Institute, Stockholm University, Stockholm S-10691, Sweden; 8 Biomedical Research and Drug Discovery Research Group, Faculty of Health Sciences, Higher Colleges of Technology, Sharjah, United Arab Emirates; 9 The Vice Chancellery, The University of Notre Dame Australia, Fremantle, Western Australia, Australia; 10 Biomedical Biotechnology Research Unit, Department of Biochemistry and Microbiology, Rhodes University, Grahamstown, South Africa; 11 Hotchkiss Brain Institute, University of Calgary, Calgary, Alberta, Canada; 12 Department of Biological Sciences, University of Pittsburgh, Pittsburgh, PA 15260, USA; 13 William Harvey Research Institute, Barts and the London School of Medicine, Queen Mary University of London, London EC1M 6BQ, United Kingdom; 14 Institute of Biochemistry and Molecular Biology, Faculty of Medicine, University of Freiburg, Freiburg, Germany; 15 Aix Marseille Univ, CNRS, BIP UMR 7281, IMM, 31 Chemin Joseph Aiguier, Marseille 13402, France; 16 Laboratoire de Microbiologie et Génétique Moléculaires (LMGM), Centre de Biologie Intégrative (CBI), Université de Toulouse, CNRS, Université Toulouse III - Paul Sabatier (UT3), Toulouse, France; 17 Department of Plant Molecular Biology, Faculty of Biology and Medicine, Lausanne University, Lausanne 1015, Switzerland; 18 Department of Pharmaceutical Chemistry and the Institute for Neurodegenerative Diseases, University of California San Francisco, San Francisco, CA 94308, USA; 19 Novo Nordisk Foundation Center for Protein Research (CPR), Faculty of Health and Medical Sciences, University of Copenhagen, Copenhagen, Denmark; 20 Department of Molecular & Clinical Cancer Medicine, Institute of Systems, Molecular and Integrative Biology, University of Liverpool, Liverpool, United Kingdom; 21 Department of Chemistry, Lafayette College, Easton, PA, USA; 22 Center for Alzheimer's and Neurodegenerative Diseases, UT Southwestern Medical Center, Dallas, TX, USA; 23 Peter O'Donnell Jr Brain Institute, UT Southwestern Medical Center, Dallas, TX, USA; 24 Leibniz Institute on Aging - Fritz Lipmann Institute and Institute of Biochemistry and Biophysics, Friedrich Schiller University Jena, Jena 07745, Germany; 25 LMU Munich, Biocenter-Cell Biology, Großhadernerstr. 2, Planegg-Martinsried 82152, Germany; 26 Australian Regenerative Medicine Institute, Monash University, Clayton, Victoria, Australia; 27 Centre for Dementia and Brain Repair at the Australian Regenerative Medicine Institute, Monash University, Melbourne, Victoria, Australia; 28 Institute of Chemistry, University of Campinas-UNICAMP, P.O. Box 6154, 13083-970 Campinas, SP, Brazil; 29 University of Cambridge, Cambridge CB2 0XY, United Kingdom; 30 Department of Biological Sciences, Indian Institute of Science Education and Research, Bhopal, Bhopal, Madhya Pradesh, India; 31 IISER Bhopal, Room Number 117, AB3, Bhopal Bypass Road, Bhopal 462066, Madhya Pradesh, India; 32 Department of Biochemistry, Rappaport Faculty of Medicine, Technion-Israel Institute of Technology, Haifa 31096, Israel; 33 Department of Molecular Biosciences, Faculty of Life Sciences, Kyoto Sangyo University, Kyoto 603-8555, Japan; 34 Department of Biomedical Sciences, University of Groningen, University Medical Center Groningen, Groningen, The Netherlands; 35 National Institute of Diabetes, Digestive, and Kidney Diseases, National Institutes of Health, Bethesda, MD 20892, USA; 36 Foundation for Polish Science, Warsaw 02-611, Poland

**Keywords:** Evolution, Hsp70 cycle, JDP

## Abstract

J-domain proteins (JDPs) are the largest family of chaperones in most organisms, but much of how they function within the network of other chaperones and protein quality control machineries is still an enigma. Here, we report on the latest findings related to JDP functions presented at a dedicated JDP workshop in Gdansk, Poland. The report does not include all (details) of what was shared and discussed at the meeting, because some of these original data have not yet been accepted for publication elsewhere or represented still preliminary observations at the time.

## Introduction to the meeting

After 2 years of COVID-related delay, the second Cell Stress Society International-sponsored meeting on J-domain proteins (JDPs) was held at the University of Gdansk, Gdansk, Poland. *Maciej Zylicz*, one of the pioneers in the JDP field of research, opened the meeting and reminded us how in the mid 1980s the eponymous DnaJ protein from *Escherichia coli* was for the first time purified and characterized biochemically using bacteriophage lambda DNA replication as a model system.^1^ The multistep reaction mediating lambda DNA replication not only allowed for the discovery that DnaJ and its partners DnaK and GrpE possess molecular chaperone activities, but also helped to uncover their functional domains: the J-domain of DnaJ as well as the ATPase and substrate binding domains of DnaK. Some of those pioneering experiments were carried out at the University of Gdansk.^2^, ^3^, ^4^ Further efforts from several laboratories demonstrated that the sequences of these chaperones are not only conserved but also their basic modes of actions are maintained across all domains of life, with specifications to serve an increasingly expanding set of protein guidance functions within cells.

## JDP evolution

As obligatory partners of heat shock protein (Hsp)70s, JDPs are present in essentially all organisms from bacteria, to both unicellular and multicellular eukaryotes, and in several viruses.^5^, ^6^ Operationally, JDPs have been divided into 3 classes according to the domains they share with the prototypic JDP, *E coli* DnaJ. In class A JDPs the N-terminal J-domain is followed by a glycine-phenylalanine rich region (G/F), 2 homologous β-sandwich domains, βSD1 and βSD2 (previously termed CTDI and CTDII), with a zinc-finger like β-hairpin inserted into βSD1, and a C-terminal helical dimerization domain. Class B JDPs show a similar domain organization with generally a longer G/F-rich region and the zinc-finger like β-hairpin missing. Class C JDPs only share the J-domain, which is not necessarily at the N-terminus but may be anywhere within the sequence.^7^, ^8^

*Pierre Genevaux* delved into a thorough analysis of bacterial and viral genomes, representing all taxonomic groups, to explore the diversity of the JDP repertoire and their relations with the different Hsp70 partners. Their analyses revealed that while DnaJ is the most broadly distributed JDP (present in 99.9% of analyzed bacterial genomes), bacterial and viral genomes constitute an unexpected reservoir of novel JDPs whose functions await exploration. In particular, class C JDPs show a clear expansion with respect to class A and B JDPs.^9^

An analysis on a broader taxonomic scale, encompassing pro- and eukaryotes, was presented by *Paolo De Los Rios*. He also highlighted the fact that class C JDPs have seen a tremendous expansion in eukaryotes, with the J-domain being genetically paired overall with more than 2,000 other domains. Remarkably, an Artificial Neural Network analysis of the J-domain sequences alone allowed us to distinguish JDPs apart based on a plethora of orthogonal criteria, such as taxonomy, localization, class (A, B, or C), and identity of the remaining polypeptide domains. A more detailed investigation revealed that the most relevant sequence positions to discriminate J-domains from each other are the ones that are in contact with Hsp70, thus suggesting that the physical interaction with this obligatory partner, and the consequence of co-evolution with its sequence, is the main driver of the sequence evolution of the JDP J-domains.^5^

An evolutionary relationship among class A and B JDPs from all 3 domains of life was a subject of research presented by *Bartlomiej Tomiczek*. Detailed, phylogenetic analyses involving class A and B JDPs from bacteria, archaea, and all sub-cellular compartments of eukaryotic cells revealed 2 unexpected findings. First, class B JDPs functioning in bacteria and in the cytosol and endoplasmic reticulum (ER) of eukaryotes evolved from class A DnaJ-like ancestors independently from each other. Thus, class B JDPs from the eukaryotic cytosol are more closely related to cytosolic class A JDPs than to bacterial class B JDPs. Second, highly divergent cytosolic JDPs involved in the suppression of amyloid fiber formation (eg, DNAJB6 and DNAJB8) evolved from a canonical class B ancestor.

In a general discussion session, the idea of a re-classification and hence revision of the nomenclature of the JDP family was further debated in light of these recent findings. Indeed, the current classification is merely operational and does not always match with genomic and evolutionary analyses. For the human JDPs, this is especially true for the 2 distinct subgroups within the current class B JDPs in which DNAJB2, -6, -7, -8 do not cluster with the other more canonical members (DNAJB1, -4, -5, -9) and where DNAJB12 and 14 cluster together with the class C DNAJC18.^5^ While it is important to reiterate these distinctions and highlight them where suitable in future publications, it was decided that changing the current nomenclature would not be a significant improvement and thus of limited value to the field.

## JDP involvement in the regulation of the heat shock response

The expression of some JDPs (albeit only a subset) is upregulated by proteotoxic stress via activation of the human heat shock transcription factor Hsf1. Hsf1 is known to be largely monomeric under non-stress conditions and upon temperature upshift, Hsf1 trimerizes and binds to the heat shock elements in promoters and enhancers of heat shock genes. *Matthias Mayer* reported on earlier findings of his group demonstrating that DNAJB1 and Hsc70 are key to this mode of regulation: they dissociate Hsf1 from DNA by monomerizing trimeric Hsf1 thereby attenuating the heat shock response.^10^ The question they next addressed is whether DNAJB1 is the only of the ∼47 JDPs that is able to assist Hsc70 in regulating Hsf1. Focusing on members of the JDP classes A and B that are believed to have general chaperone function and reside in the nuclear-cytoplasmic compartment, they found that only class B JDPs could cooperate with Hsc70 in dissociating Hsf1 from DNA, although all JDPs tested were able to stimulate the ATPase activity of Hsc70. Moreover, class A JDPs were even more efficient in assisting refolding of a model substrate, heat-denatured firefly luciferase, than class B JDPs. Therefore, there must be a special property of class B JDPs that is absent in class A JDPs and that is necessary for Hsf1 monomerization, but this seems to be less important for their general chaperone activity.

## The Hsp70 cycle—co-translational folding

Whereas Hsps were originally discovered in response to heat shock (that causes protein unfolding) and whilst JDP functions were originally pioneered for their involvement in (phage) replication, one of their dominant functions relates to co-translational folding. Translation is the first step in a protein’s life cycle and *Sabine Rospert* described features of the mechanism by which the ribosome-associated complex (RAC) mediates the co-translational association of Hsp70 with nascent polypeptide chains. RAC is a heterodimer consisting of Zuo1, a ribosome-associated JDP, and Ssz1, a non-canonical Hsp70, which does not hydrolyze ATP. RAC is established to act as a J-domain partner of the ribosome-bound yeast Hsp70-homolog Ssb. Data were presented to show that Zuo1 and Ssz1 interact with nascent chains prior to Ssb. Zuo1 and Ssz1 thereby mediate efficient binding of nascent chains to Hsp70 Ssb.^11^ Zuo1 associates with nascent chains when Ssz1 is absent, so it independently binds to nascent chains. Interestingly, structural studies indicate that within the RAC heterodimer, the very N-terminus of Zuo1 binds to the Ssz1 substrate binding domains as a pseudo-substrate. Zuo1 thereby competes with nascent chain binding to Ssz1.^12^ These data describe the reaction cycle whereby the initial interaction of Hsp70 with nascent chains is regulated by a JDP on ribosomes.

*Bernd Bukau* next reported on the use of ribosome profiling to dissect, in a genome-wide manner, the co-translational action of chaperones in protein folding steps that depend on the length of growing nascent polypeptide chains.^13^, ^14^ The nascent chain interactome of yeast RAC was first described and indicated that single or multiple binding events of RAC to ribosome-nascent chain complexes (RNCs) occur. These data indicate that (1) RAC does not continuously bind to RNCs, (2) RAC can bind/rebind throughout protein synthesis and (3) RAC relies on recognition of sequence features within the nascent chains. Thus, RNCs seem to be under constant surveillance by RAC with the sequence of individual polypeptides, thereby dictating the rate of RAC cycling on and off during nascent chain elongation. Together, these data illustrate mechanisms of action for JDPs and Hsp70s at the earliest stage of a protein’s life.

## Biogenesis of mitochondrial proteins

Most mitochondrial proteins are synthesized on cytosolic ribosomes and imported into mitochondria in a post-translational reaction. This process comprises several steps: first proteins must be delivered to mitochondria. Next, they must be targeted to the mitochondrial translocon, and, in the case of mitochondrial matrix proteins, they have to be pulled into the mitochondria by the import motor. Hsp70 systems, both cytosolic and mitochondrial, are involved in several of these steps, with their participation being dictated by specific members of the JDP family. *Doron Rapaport* explained how proteins of the mitochondrial outer membrane are delivered to mitochondria by Hsp70 and Hsp90 chaperones, which bind hydrophobic transmembrane segments that are exposed by membrane proteins that are not yet inserted in the membrane. In yeast, this process is crucially regulated by the abundant and generic cytosolic JDPs, Ydj1, and Sis1, which belong to classes A and B, respectively. Importantly, interfering with these interactions disrupts the biogenesis of mitochondrial outer membrane proteins to various extents.^15^, ^16^

*Johannes Herrmann* highlighted the role of the ER in facilitating the targeting of some newly synthesized mitochondrial proteins, in particular hydrophobic proteins of the mitochondrial inner membrane like Oxa1, to the organelles. These proteins might first bind to the ER membrane, and from there they diffuse to contact points between the ER and mitochondria where the mitochondrial outer membrane protein, Tom70, which is part of the translocation channel, is also localized. An ER-membrane localized, class C JDP, Djp1, was found to be necessary for the correct insertion of Oxa1 in the inner membrane, a process that has been called ER-SURF. Deletion of Djp1 results in the accumulation of Oxa1 in the cytosol. While the precise molecular mechanism of the Djp1 action, and whether its function is Hsp70-dependent, has not been elucidated, these findings shed light on the complexity of the biogenesis of mitochondrial proteins, with a crucial role for a JDP.^17^

After being targeted to the mitochondrial surface, many proteins must be further imported into the mitochondrial matrix through the mitochondrial import pore. Although this import pathway has been studied for almost three decades, new discoveries emphasize its great complexity. *Dejana Mokranjac* reviewed the central role of the highly specialized JDP system involved in this process. It consists of a specialized class C JDP and a paralogous adapter protein possessing a J-like domain with a corrupted HPD triad. This J-like adapter recruits JDP to the import site and also appears to control its ability to activate Hsp70. Alternative models of the mtHsp70 cycle within the import motor were discussed.^18^

## ER-resident JDPs

Like mitochondria, the ER has its own repertoire of JDPs that collaborate with the ER-specific Hsp70 known as BiP (or HSPA5). Hsp70 family members are known to be subject to regulation by post-translational modification.^19^ *David Ron* presented data on the post-translational AMPylation of BiP, a modification that his group previously found to modify interactions with ER-luminal JDPs.^20^ BiP AMPylation is a metazoan-specific adaptation that impacts organismal biology.^21^, ^22^ David showed that, in animals, BiP undergoes reversible AMPylation and deAMPylation at Thr518, a highly conserved residue within the substrate binding domain of BiP. This modification stalls BiP in its ATPase domain-docked state to which the effector J-domains of co-chaperones interact unproductively. The bifunctional ER-localized enzyme FICD is responsible for Thr518-AMPylation and interestingly FICD mutations are associated with loss of neuronal fitness. So, non-productive BiP-J-domain interactions could underlie these phenotypes.

*Ryo Ushioda* introduced functions of ER-luminal DNAJC10/ERdj5 and BiP in maintaining ER homeostasis. ERdj5 contains 6 thioredoxin-like domains and cleaves disulfide bridges in misfolded proteins, thereby contributing to efficient retro-translocation during ER-associated proteasomal degradation (ERAD).^23^ ERdj5 also acts in the lumen to regulate calcium pumps and channels in the ER membrane.^24^ ERdj5 exerts the reciprocal regulatory function for disulfide-containing inositol 1,4,5-trisphosphate receptors and sarcoplasmic/endoplasmic reticulum Ca^2+^-ATPase 2b by sensing the ER luminal calcium concentration.^24^, ^25^ Thus, ERdj5 is a multi-functional JDP acting in ERAD and ER-calcium homeostasis.

There is a sub-family of ER-transmembrane JDPs containing cytosolic J-domain that includes DNAJB12, which recruits Hsp70 function on the cytoplasmic face of the ER.^6^ *Doug Cyr* presented evidence suggesting that DNAJB12 plays an essential role in organismal biology by functioning with Hsp70 to prevent the accumulation of toxic intermediates of misfolded membrane proteins.^26^, ^27^ DNAJB12, which has a J-domain in the cytosol and a putative calcium-binding domain in the ER lumen that are connected by a transmembrane domain, recruits Hsp70 to function in the selection of globally misfolded membrane proteins for ubiquitination and degradation by ERAD. DNAJB12 and Hsp70 can also target pools of kinetically trapped and ERAD-resistant membrane proteins for selective degradation via ER-associated autophagy. Hence, DNAJB12 is critical for the quality control of membrane proteins and ER-homeostasis. In addition, loss of DNAJB12 predisposes cells to apoptotic death^28^ and DNAJB12's role in quality control of membrane proteins is critical for ER-homeostasis.

## JDP—Hsp70 interaction and regulation

Given the extent and diversity of the JDP family, one wonders whether all of them interact with and regulate the Hsp70 polypeptide binding and release cycle in a similar manner or not. In eukaryotic cells, most nuclear-cytosolic Hsp70s contain a conserved EEVD motif at the C-terminus which is implicated as a binding site for cochaperones containing tetratricopeptide repeats. The EEVD motif is also implicated in influencing interactions of the Hsp70s C-termini with the polypeptide binding domain. Interestingly, the J-domain of the yeast class B JDP Sis1 has also been reported to bind the EEVD motif, suggesting a novel mechanism for cooperation between Hsp70 by this class of JDPs.^29^, ^30^ The same is true for mammalian class B JDPs.^31^ To explore the nature of Sis1 interactions with EEVD the group of *Carlos Ramos* reported on recent NMR studies.^32^ He presented NMR chemical shift assignments for ^1^H, ^15^N, and ^13^C nuclei of the backbone and side chains of the J-domain of Sis1, complexed with the C-terminal EEVD motif of Hsp70. The data revealed information on the structure and backbone dynamics that add significantly to the understanding of the J-domain-Hsp70-EEVD mechanism of interaction.

The canonical Hsp70 cycle as it is generally presented in reviews was critically discussed at the meeting. The cycle is usually depicted that JDPs first bind to the polypeptide substrates, recruit Hsp70, and next hand over the substrate to Hsp70 via stimulation of its ATPase (by both the J-domain of the JDPs and substrates) upon which the JDP is released from the substrate-Hsp70 complex. Whereas this maybe consistent with some JDP-Hsp70 functionalities—as in the biogenesis of iron-sulfur clusters^33^—more and more data now show that JDPs are not always released from the substrate upon Hsp70 binding and that complexes with substrates exist where both JDPs and (multiple) Hsp70s are substrate-bound, for example, as in clathrin uncoating^34^, ^35^, ^36^, ^37^, ^38^, ^39^, ^40^, ^41^, ^42^, ^43^, ^44^, ^45^, ^46^ or amyloid disaggregation.^37^ What actually then drives substrate release of JDPs remains to be elucidated, but it was speculated to require forces evoked by entropic pulling of (one or multiple) Hsp70s to change the configuration of the JDP-binding site at the substrate from a high to low-affinity state.^38^ This would imply that, in some cases, the mechanism would be more like a clip-off type rather than a hand-over. It would also explain why Hsp70s seem dispensable for some JDP activities at all, for example, in holding-like functions based on low-affinity substrate interactions.

In the same general discussion, we also talked about the role of the auto-inhibition that is seen for at least some JDPs, where the Hsp70-interacting surface of the helix 2/helix 3 hairpin of the J-domain is shielded by helix 5 and/or 6, which is present in the G/F region.^31^, ^39^, ^40^ Besides stabilizing the unstructured regions of these JDPs, the auto-inhibition might serve to prevent unproductive Hsp70 recruitment, that is, to those JDPs that are not substrates bound. The latter would imply that JDP substrate binding would unlock this auto-inhibition, but this model currently lacks experimental evidence. In addition, the EEVD of Hsp70s might play a role in unlocking some JDPs^29^, ^31^ which would extend the role of the EEVD in Hsp70 beyond increasing the Hsp70 affinity for JDPs.

## Class C JDPs

As discussed above, mitochondrial biogenesis requires the contribution of each 1 of the 3 JDP classes. While classes A and B have been extensively studied, in particular in connection with their role in protein refolding and disaggregation, class C JDPs have attracted less attention, with few well-known exceptions, possibly owing to their staggering diversity within and between organisms. This is nonetheless changing due to increased recognition that class C JDPs are critical for a myriad of different cellular processes performed by Hsp70 systems. While studies of class C JDPs are in most cases devoted to specific instances, often because of their specific biological relevance, in the future the collection of observations will likely provide a more fundamental understanding of the triage role of this highly divergent class of JDPs.

Simple architecture and well-characterized interactions with Hsp70s and clients of some class C JDPs give us the possibility of using them to address fundamental questions about JDPs' mechanism of action. *Jaroslaw Marszalek* and co-workers exploited the specialized class C JDP involved in the biogenesis of iron-sulfur cluster-containing proteins in bacteria (HscB) and in mitochondria (Hsc20) to elucidate how the client-bound JDP recruits a Hsp70 partner. In this system, a structurally rigid JDP-client complex recruits Hsp70 via precise positioning of the J-domain and client at their respective interaction sites with Hsp70- resulting in functionally high-affinity interactions that arise from avidity. While the high degree of avidity of this specialized system may seem unusual, however, functionally important avidity driven by JDP-client interactions is likely sufficient to explain synergistic ATPase stimulation and efficient substrate trapping in many JDP/Hsp70 systems.^41^

*Olivier Genest* and *Sébastien Dementin* discussed the atcJABC operon of *Shewanella oneidensis*, a bacterium whose ability to absorb and reduce heavy metals suggests the potential for applications in, for example, wastewater treatment. The AtcJ protein is the shortest class C JDP studied to date, comprising the J-domain and a short, 21-residue C-terminal extension only. AtcJ is required for bacterial growth at low temperature, through a mechanism that has still to be elucidated.^42^ Because Atc proteins are conserved in several environmental proteobacteria, they are likely to confer new, still unresolved biological functions to DnaK (bacterial Hsp70) for bacterial adaptation to stresses.^43^

*Gregory L. Blatch* provided an intriguing account of the class C JDPs of *Plasmodium falciparum* (PfJDPs). More than a third of the PfJDPs (18/49) are exported with the goal to re-functionalize both the parasite and host Hsp70s, thereby transforming the infected host cells into vehicles of pathology. The increasing drug resistance of the malarial parasite has triggered the need to develop novel therapeutics, and the PfJDP-Hsp70 interaction interface may represent a potential novel drug target. Preliminary virtual screening of a drug repurposing library identified some candidate compounds.^44^

Compared to prokaryotes, eukaryotes, and in particular multicellular organisms, have seen a remarkable expansion of their JDP repertoire,^45^ driven in particular by an increasing number of class C members, with plants outnumbering all other species: *Arabidopsis thaliana* encodes for more than a hundred JDPs, most of which are class C. *Pierre Goloubinoff* described his recent findings about the molecular mechanism of heat sensing in this model organism, which starts at the membrane through the heat-induced activation of the cyclic nucleotide-gated channel 2. By yeast 2-hybrid screen, his group identified a plant-specific class C JDP that binds cyclic nucleotide-gated channel 2. Mutations in these JDPs affected the plant’s ability to produce Hsps in response to an initial heat shock. Strikingly, these mutants showed an even less efficient ability to produce Hsps in response to subsequent heat shocks. These results suggested a role for Hsp70s, guided by these plant-specific JDPs, in recharging the heat-depolarized thermo-sensory channels in the plasma membrane.

Human class C JDPs have also recently been the focus of several studies, highlighting the participation of Hsp70 chaperones in a plethora of cellular processes. *Colin Hammond* showed that different Hsp70 isoforms participate in histone maintenance. In particular, he used structure-guided and functional proteomics assays to reveal that DNAJC9 is a key JDP that uses its N-terminal J-domain to recruit several Hsp70s to histones, integrating these chaperones into the DNA-replication and transcription-coupled nucleosome assembly pathways.^46^ This discovery is also linked to original findings showing that prokaryotic JDPs are engaged in DNA-related metabolism as was highlighted by Maciej Zylicz.

Chaperoning of misfolded proteins is often associated with the JDP-driven action of Hsp70s, not only through protein repair (protein disaggregation, unfolding and, upon release, refolding to the native state) or protein degradation, but also through delivery to different vesicles. In 2 separate talks, *Janice Braun* and *Yihong Ye* presented new results on DNAJC5 (CSPα-cysteine string protein). Depending on its membrane localization, whether at the Golgi or at the lysosome, DNAJC5-mediated vesicle export into the extracellular space or endolysosomes eliminate a diverse set of misfolded proteins including misfolded ⍺-synuclein, TDP-43, Tau, SOD-1 and huntingtin among others. Yet, the full range of DNAJC5 secretion substrates is unknown.^47^, ^48^ Mutations within DNAJC5 are known to lead to adult-onset neuronal ceroid lipofuscinosis a rapidly developing neurodegenerative disease.^49^ Nevertheless, the precise relation between DNAJC5, its involvement in the secretion of misfolded proteins, and the disease is still unclear, as DNAJC5-deficient mice develop neurodegeneration whereas in vitro, adult-onset neuronal ceroid lipofuscinosis-associated DNAJC5 mutations inhibit the secretion of misfolded proteins.

Sacsin (DNAJC29) is one of the longest human proteins and the longest class C JDP. Some of its mutations are related to Autosomal Recessive Spastic Ataxia of Charlevoix-Saguenay, a neurodegenerative disorder with a neurodevelopmental component. *Paul Chapple* and co-workers undertook a comprehensive molecular characterization of DNAJC29 knockout cells and used structural modeling to investigate its chaperone function. He discussed results that suggest DNAJC29 is an ATP-dependent molecular chaperone required for trafficking and localization of synaptic adhesion proteins.^50^

## Protein aggregation related to acute stress and chronic diseases

An important issue in the field is still how Hsp70s and JDPs deal with unfolded or misfolded proteins as they arise after acute stress conditions such as heat shock (which is how Hsps were discovered) or when mutant, disease-related proteins are expressed that are associated with the formation of disordered protein aggregates or ordered, amyloid assemblies. Open questions include which JDPs are involved, how and where do they bind and where (both from the perspective of JDP and substrate side), if and how Hsp70s are involved and recruited, and how the assemblies are processed.

*Rina Rosenzweig* described a new binding site in class A JDPs that can recognize the increase in dynamics and transient breakage of hydrogen bonds in β-sheet-rich proteins. Through this site, class A JDPs can bind and stabilize these proteins at the initial stages of protein misfolding. Interestingly, once bound to such misfolded proteins, class A JDPs assemble into large oligomeric particles, thus sequestering these clients. This mode of sequestration of misfolding clients is reminiscent of the mode of action of small Hsps.^51^, ^52^, ^53^ Rosenzweig and coworkers propose that these class A JDP-client sequestration complexes protect destabilized β-sheet-rich proteins from more extensive misfolding and formation of large aggregates during stress. Once the stress is alleviated, these complexes can be disassembled by the Hsp70 chaperones, releasing the proteins into solution that next rapidly can fold into their native states.

*Harm Kampinga* showed a comparable paradigm for DNAJB6. This class B JDP, which delays amyloidogenesis of multiple proteins (including polyQ proteins) associates with early condensates of polyQ and prevents their transition from a liquid to an amyloid state,^54^ again mimicking an Hsp70 independent “holdase“ action of small Hsps. Using an inducible-aggregation system, Kampinga and coworkers provided evidence that such an action may next support an Hsp70-dependent fragmentation and autophagic clearance of the substrates. In addition, he reported on their recent findings that DNAJB6 is involved in the biogenesis of nuclear pore complexes,^55^ which seems associated with this holding function of DNAJB6 to substrates such as the intrinsically disordered FG-repeats, which are integral components of the nuclear pore complex, thus preventing their transition from a soluble to a solid state. A prime feature of the absence of DNAJB6 is impaired to nucleocytoplasmic shuttling, expanding the repertoire of important cell biological functions that JDPs are engaged in.

Another study on the suppression of aggregation of neurodegenerative disease-associated proteins by JDPs was reported by *Reut Shalgi*. She focused on a FUS mutant that is related to amyotrophic lateral sclerosis.^56^ A quantitative chaperone overexpression screen, not only confirmed the broad anti-amyloidogenic capacities of DNAJB6 and DNAJB8 but also identified DNAJB12 and DNAJB14 as novel modulators of FUS aggregation.^57^ DNAJB12 and DNAJB14 are 2 rather unexplored homologous JDPs with an N-terminal α-helical domain followed by the J-domain, a linker region, a transmembrane domain, and a C-terminal α/β-domain of unknown function. Shalgi pointed out that shorter isoforms of both JDPs occur naturally. The short form of DNAJB12 lacks the N-terminal α-helical domain and the J-domain, whereas the short form of DNAJB14 only consists of the N-terminal α-helical domain and lacks the J-domain, the transmembrane domain, and the C-terminal α/β-domain. The full-length isoforms of DNAJB12 and DNAJB14 form a complex that potently inhibited mutant FUS aggregation in an Hsp70-dependent manner. In contrast, the short isoforms of DNAJB12 and DNAJB14, lacking the J domain and thereby their ability to cooperate with Hsp70, do not form a complex with each other and are unable to suppress aggregation of mutant FUS. Surprisingly, the isoforms of DNAJB12 had opposing effects on the aggregation of polyQ huntingtin as compared to amyotrophic lateral sclerosis-related FUS. DNAJB12-short significantly inhibited, while the full-length isoform of DNAJB12 significantly enhanced HTT-polyQ aggregation.^57^ These findings highlight 3 contributing features to Hsp70-JDP network complexity: JDP isoforms, JDP heterodimers, and Hsp70-dependent and Hsp70-independent functions of JDPs.

What happens if protein aggregates are already formed? How classes A and class B JDPs differ in recognition and targeting of Hsp70s to amorphic aggregates was studied in detail by *Krzysztof Liberek* and colleagues using the yeast *Saccharomyces cerevisiae* Hsp70 chaperone Ssa1 and its class A JDP Ydj1 and class B JDP Sis1 as a model system. With real-time biochemical tools, they showed that Sis1 attracts more Hsp70 to amorphic aggregates than Ydj1.^58^ Presence of Sse1, the nucleotide exchange factor, increased both the association of Hsp70 and JDPs with aggregates and subsequent disaggregation and refolding. The positive effect of Sse1 was more pronounced when Sis1 was involved and was dependent on Sis1’s ability to bind to the EEVD motif at the C-terminus of Hsp70 Ssa1, a trait that is specific to class B JDPs and is absent in class A JDPs.^29^, ^31^

In bacteria, plants, and fungi, JDPs and Hsp70s have been shown to cooperate with Hsp100 AAA+ disaggregase for protein disaggregation, mostly resulting in (partial) functional recovery of aggregated proteins.^59^ The canonical Hsp100 AAA+ disaggregases, however, seem to be absent in the metazoan cytosol. For efficient solubilization of amorphous protein aggregates in metazoa, it was shown previously that this is accomplished by hybrid formation of class A and class B JDPs that next interact with Hsp70 and the Hsp110 nucleotide exchange factor.^60^ If and how the assembly of this protein disaggregases is arranged in living cells, however, has remained unanswered. *Nadinath Nillegoda* reported that they, for the first time, selectively tracked the disaggregases in human cells. In fact, they demonstrated the hybrid DNAJA1-DNAJB1-Hsp70 disaggregases actually act in tandem with yet another solubilization system, comprising of the AAA+ protein VCP, to solubilize different populations of heat-induced aggregates in space and time. Importantly, evidence was provided showing that the assembly of the Hsp70-DNAJA1-DNAJB1 disaggregase is severely hampered in human cells that undergo replicative aging, leading to increased occurrences of pathological protein aggregation.^61^

How are pre-existing structured amyloid aggregates, which likely present a more formidable barrier, handled? Recent data showed that DNAJB1 together with Hsp70s and its nucleotide exchange factors Hsp110 (HSPH1 or Apg2) can disaggregate fibrils formed by alpha-synuclein.^37^ *Janine Kirstein* had published earlier that the same 3 proteins could reverse fibrils formed by mutant huntingtin.^62^ To do so, DNAJB1 binds with the hinge region between the C-terminal domains 1 and 2 to the prolines that flank the C-terminal end of the polyglutamine stretch in huntingtin. In fact, the interaction could be narrowed down to a single amino acid (histidine-244) within DNAJB1 that forms a hydrogen bond with a glutamate residue in the hinge region, which forms a binding platform for the specific interaction with HTT. Mutation of histidine-244 abrogated any chaperone activity toward huntingtin but was found to have no effect on other protein substrates tested. These data highlight how a generalist like DNAJB1 can use distinct binding sites to provide specificity for their protein substrates. Notably, Hsp70 (HSPA8) was found to interact with mutant huntingtin as well, but only in the presence of DNAJB1, and also bound to the same proline-rich domain of HTT.^63^

A question that also emerged lately is whether or not breaking down amyloid fibrils is beneficial. For many neurodegeneration-associated amyloid fibrils, it has been shown that they can spread in a prion-like manner from cell to cell. Hsp70s with their JDP companions might contribute to spreading through the fragmentation of amyloid fibrils into small oligomers that could act as seeds.^64^, ^65^ It is therefore of prime importance to elucidate if and how chaperones in general and JDPs in particular could modulate adequate disposal of seeds, their growth, and their transmission. In this regard, *Lukasz Joachimiak* reported on the discovery of 2 JDPs that modify Tau seeding in a cellular system. One of them, DNAJC7/Tpr2, seems to bind natively folded Tau and prevents aggregation in a seed-independent manner. In contrast, DNAJB8 prefers tau seeds compared to inert aggregation-resistant monomers. DNAJB8 forms higher order assemblies, and a short phenylalanine-based motif was identified that is necessary for DNAJB8 oligomerization. Mutating the respective phenylalanines to serine residues results in monomeric forms of DNAJB8 that retain not only its activity to prevent tau seeding but also the previously discovered activity of delaying polyQ aggregation.^66^

How are chaperoned seeds next handled? As refolding is unlikely to be successful in this case, degradation would be the preferred option to prevent potentially toxic effects on the cells after all. Indeed, it was shown previously that Hsp70 is closely integrated into the process of degrading misfolded proteins and hereto acts by maintaining misfolded proteins in a soluble state and targeting them to proteolysis by the ubiquitin-proteasome system (UPS)- and autophagy-dependent proteolysis.^67^ Recent comprehensive analysis of peptides that Hsp70 binds show that they function as UPS degrons, suggesting close integration of Hsp70 and UPS at the level of substrate recognition.^68^ The same appears to be true also for the autophagic removal of protein aggregates. *Claes Andréasson* reported on how yeast cells remove stable amyloid aggregates using an Hsp70-dependent autophagy pathway. Hsp70 and JDPs appear to function as a recognition component not only in the UPS but also when targeting persistent protein aggregates for autophagy. This targeting is linked to the NEF-mediated substrate release from Hsp70.

## Yeast prions

The model organism *S cerevisiae* (yeast) is particularly suitable for investigations of the assembly, disassembly, and, more generally, control of amyloid-like structures formed by endogenous prion proteins. Yeast contains more than 10 natural prions, the best studied of which are [URE3], [PSI^+^], and [PIN^+^].^69^, ^70^ *Justin Hines* reported on his investigations on the influence of JDPs on prion propagation in *S cerevisiae* and which and how JDPs support the action of high levels of the AAA+ disaggregase Hsp104 in “curing” yeast cells of prions, that is, leading from prion-infected cells to prion-free progeny by several different mechanisms. Of the 13 yeast nuclear-cytoplasmic JDPs, only the depletion of the class A JDP Apj1 and the class B JDP Sis1 impair Hsp104-mediated curing of yeast cells, and overexpression of Apj1 and Sis1 enhance Hsp104-mediated curing. In contrast, overexpression of Ydj1 prevents Hsp104-mediated curing. A truncation variant of Sis1 (Sis1(1-121)), that only contains the J-domain and the N-terminal fragment of G/F rich region but not the G/M rich region nor the C-terminal client binding and dimerization domains, was able to support yeast growth and to maintain strong but not weak [PSI^+^] variants (*strong* and *weak* characterizing the strength of the prion phenotype). However, Sis1(1-121) did not allow curing of the strong [PSI^+^] prion by overexpression of Hsp104.^71^ Similarly, truncation constructs of Apj1 containing the N-terminal J-domain and the glycine/phenylalanine/glutamine/serine-rich region (Apj1(1-161)) allowed curing of the strong [PSI^+^] and replacement of the J-domain and glycine/phenylalanine/glutamine/serine rich-region of Apj1 by the J-domain and G/F-region of Ydj1 blocked Hsp104-mediated curing of the prion. These data indicate that the key functions important for assisting Hsp104-mediated curing reside in this region of the JDP.^72^

*Dan Masison* provided evidence that human DNAJB6 cures yeast cells of toxic, structurally similar [URE3] and weak [PSI^+^] prions. In contrast, DNAJB6 does not cure yeast cells of strong [PSI^+^] prions but protects them from prion-associated toxicity. So, DNAJB6 anti-amyloid activity is limited by amyloid structure, not the protein composing it. In addition, DNAJB6-mediated protection from amyloid toxicity seems to be mechanistically different from the protection provided by the yeast class B JDP Sis1. DNAJB6 also protects yeast cells from the toxicity of Huntingtin (Htt)-related polyglutamine (polyQ). DNAJB6 sequesters and colocalizes with the polyQ aggregates in insoluble protein deposit. The insoluble protein deposit was previously found as a perivacuolar site where terminally aggregated proteins like Htt-Q103 and the yeast prion Rnq1 accumulate in an immobile state.^73^, ^74^ Protection from toxicity needs the C-terminal domain of DNAJB6 and colocalization with dispersed polyQ aggregates, but not the sequestration of polyQ aggregates. In contrast, the sequestration of polyQ aggregates by DNAJB6 requires interaction with Hsp70, whereas the protective effect of DNAJB6 seems to be Hsp70-independent. Therefore, DNAJB6-mediated protection from polyQ toxicity and sequestration of polyQ aggregates are separable: DNAJB6 cooperation with Hsp70 is required for sequestration of polyQ aggregates, but sequestration alone is insufficient to protect cells.

Due to the high conservation of the chaperone systems, yeast is also suitable for testing the functionality of proteins from other organisms, such as plants and humans, on yeast prions. *Chandan Sahi* reported the identification of 8 class B JDPs in *A. thaliana* that are homologs of yeast Sis1 and that colocalize with the AAA+ disaggregase Hsp101 (the Hsp104/ClpB homolog) to heat-induced aggregates. Sahi provided evidence that the inactivation of one of the JDPs resulted in a significant reduction of acquired thermotolerance. Six of the JDPs were able to replace the essential Sis1 in yeast and supported [PSI^+^] propagation, suggesting that they could also assist remodeling of amyloid aggregates in plants, thereby potentially working as epigenetic modifiers of plant growth and development.^75^

## Drugs modulating JDP-Hsp70 activity

The myriad of JDP-Hsp70 pairs in eukaryotic cells play both general and specific roles in cellular physiology, so pharmacological modulation of these functions could be of therapeutic interest. *Jeff Brodsky* presented on the development of a series of inhibitors that specifically blunt or enhance the ability of JDPs to activate the Hsp70 ATPase activity.^76^, ^77^ Specifically, these molecules bind at the interface of the Hsp70 and the JDP to either promote or block the protein-protein interaction.^78^ He showed that in cellular models that recapitulate α-synuclein and huntingtin aggregation, a lead agonist was able to reduce the accumulation of cellular aggregates.^79^ *Jason Gestwicki* reported on his progress in designing drugs that can even interfere with specific JDP-Hsp70 interactions as well as on his design of engineered J-domains. It is yet too early to state if any of these will have therapeutic potential, but it is clear that these efforts will provide the field with novel tools for both biochemical experiments and modulating the function of JDPs and Hsp70s in complex cellular systems.

## Hsp70-independent functions of JDPs

For Hsp70 to function, it has been long proposed that at minimum 1 JDP and most commonly 1 nucleotide exchange factor was needed. Inversely, however, data are emerging to suggest that JDPs have functional roles beyond their partnership with Hsp70s. In fact, the binding of JDPs to substrates does not require Hsp70 and, as stated above, provides some of them with a chaperone-like activity that resembles that of small Hsps. Whether this suffices to lead to productive effects on their substrates remains elusive and, in many cases, still requires transfer to, or clipping by, Hsp70s. However, there are other new data emerging that JDPs do indeed exhibit Hsp70-independent functions. One of these was presented by *Johannes Buchner* and concerns a novel coupling factor, NudC. NudC was identified in a CRISPR screen in collaboration with the lab of Martin Kampmann.^80^ The results show that NudC, like Hsp70/Hsp90 coupling factors, affects the folding and activation of the Glucocorticoid receptor (GR). Whereas GR biogenesis is generally dependent on a sequentially coupled activity of Hsp70 and Hsp90, the surprising finding was that NudC binds not only to Hsp90 but also to JDPs. This allows for the formation of a trimeric NudC-JDP-GR complex in which GR is directly transferred from the JDP to Hsp90, without the involvement of Hsp70.

## Concluding remarks

**Fig. 1:**
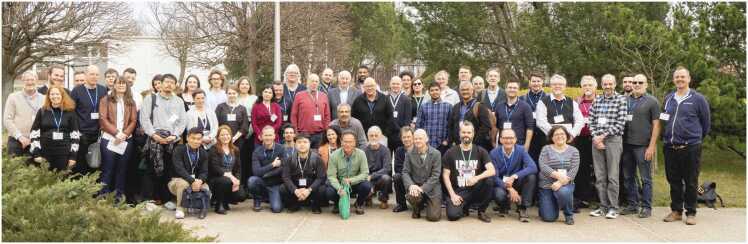
Group photo.

This second specialized meeting on JDPs highlighted many novel discoveries in the field, pointing toward a much more central role of JDPs in protein quality control than previously assumed. Even though JDPs are the largest family of chaperones in most species, the number of their representatives in each organism is nonetheless limited, and thus, as the data show, individual JDP members show great flexibility in handling multiple different substrates with great versatility to orchestrate cellular protein quality control at many different levels, with relevance to many different major cellular functions. New modes of action, both with and without engagement of Hsp70, are being discovered and JDP-targeting drugs are on the horizon as research tools and maybe as future therapeutics for diseases in which protein quality control is impaired or overwhelmed (Fig. 1).

## Data Availability Statement

No data was used for the research described in the article.

## Funding information

The organizers would like to thank the Cell Stress Society International for their generous financial support of the workshop. We also thank the Rector of the University of Gdansk, the Dean of the Intercollegiate Faculty of Biotechnology, University of Gdansk and Medical University of Gdansk for financial and organizational support. We thank the Journal of Biological Chemistry for financial support.

## Declaration of interests

The authors declare that they have no known competing financial interests or personal relationships that could have appeared to influence the work reported in this paper.
